# A novel temperate phage from *Alicyclobacillus*: first evidence in this genus of genomic identity to a *sigK*-integrated prophage

**DOI:** 10.1128/spectrum.03747-25

**Published:** 2026-04-03

**Authors:** Inês Carvalho Leonardo, Helena Ferreira, Ana Patrícia Quendera, Maria Teresa Barreto Crespo, Carlos São-José, Frédéric Bustos Gaspar

**Affiliations:** 1 iBET, Instituto de Biologia Experimental e Tecnológica 449442, Oeiras, Portugal; 2 Instituto de Tecnologia Química e Biológica António Xavier, Universidade Nova de Lisboa 98819 , Oeiras, Portugal; 3 Research Institute for Medicines (iMed.ULisboa), Faculdade de Farmácia, Universidade de Lisboa 37809, Lisboa, Portugal; Universidad Nacional Autonoma de Mexico-Campus Morelos, Cuernavaca, Mexico

**Keywords:** temperate phages, thermoacidophilic spore-forming bacteria, *Alicyclobacillus* spoilage, phage-sporulation interaction, biocontrol

## Abstract

Bacteriophages are increasingly explored as clean-label, environmentally friendly tools for sustainable food processing. Several phage-based products have been approved for agri-food applications, all of which rely on strictly lytic phages to ensure safety, regulatory compliance, and rapid bactericidal action. Despite its industrial relevance, only two phages targeting the genus *Alicyclobacillus*, thermoacidophilic spore-formers responsible for fruit product spoilage, have been reported to date, leaving a very limited arsenal for their control. This study reports the *Alicyclobacillus* phage MMB025, infecting *Alicyclobacillus acidoterrestris*, a species associated with spoilage. Transmission electron microscopy revealed a contractile-tailed morphotype, and host range assays demonstrated specificity for a narrow subset of *Alicyclobacillus* isolates. MMB025 displayed stability across a wide pH range (3–12) and moderate heat (≤60°C). Hybrid sequencing identified a 105 kb dsDNA genome encoding structural, replication, and lysogeny modules, the latter indicating a temperate lifestyle. Consistently, the phage genome showed 99.99% identity to a chromosomal region of *A. acidoterrestris* DSM 3922^T^, most likely corresponding to MMB025 prophage state. Genome mapping showed prophage integration within the sporulation sigma factor gene *sigK*, reminiscent of the *Bacillus subtilis skin* element, suggesting a novel phage-sporulation interaction in *Alicyclobacillus*. Despite the presence of lysogeny-related features, bioinformatic lifestyle predictions were inconclusive, emphasizing current limitations of *in silico* classification. Although temperate phages like MMB025 are not ideal for direct biocontrol, the genome encodes putative lysins and enzymes with potential for spoilage mitigation. This study contributes to expanding the still-limited diversity of *Alicyclobacillus* phages and provides new insights into phage-host interactions in thermoacidophilic spore-forming bacteria.

**IMPORTANCE:**

The genus *Alicyclobacillus* remains a persistent problem for the fruit juice industry, as its thermoacidophilic nature leads to spoilage events that cause product recalls, loss of consumer confidence, and significant economic losses. Yet remarkably little is known about the phages infecting these bacteria, with only two described to date. This study reports the first infectious phage with genomic identity to a prophage in the genus, the *Alicyclobacillus* phage MMB025. This finding broadens the extremely limited record of *Alicyclobacillus* phages and reveals integration within the sporulation sigma factor *sigK*, suggesting a novel link between prophage activity and sporulation regulation in *Alicyclobacillus*. By combining stability assays, host range testing, and genome analysis, MMB025 represents both a valuable model for phage-spore-former biology and a genetic resource for candidate lysins with potential applications in food preservation.

## INTRODUCTION

In the fruit juice industry, alternative preservation strategies to eliminate the spoilage bacteria *Alicyclobacillus* (ACB) have been extensively explored. These gram-positive, non-pathogenic bacteria produce off-flavors and odors described as medicinal, disinfectant-like, and cheesy, compromising product quality and leading to food waste (1). ACBs are thermoacidophilic spore-forming bacteria that can survive the acidic conditions commonly used in fruit juice production. Also, their ability to form highly heat-resistant spores allows them to withstand standard pasteurization processes used in the industry. Due to their resilience and potential to cause spoilage, there is a critical need for effective alternative preservation strategies to reduce economic losses and maintain juice quality (2–4).

The increasing demand from consumers for healthy eating, clean-label products, and sustainable food practices has driven interest in environmentally friendly preservation methods. Among these, bacteriophage-based treatments have emerged as a promising strategy that aligns with the principles of green, sustainable food protection technologies. Bacteriophages (or simply “phages”), which exclusively target and infect bacteria, can be used to control both food-borne pathogenic and spoilage bacteria, offering advantages compared to traditional chemical and physical treatments. Phages have high specificity, providing a targeted approach to eliminate harmful bacteria without disrupting beneficial microbial communities. Additionally, phages can be directly applied to food products, as they are biodegradable, leave no toxic residues, and do not alter the organoleptic properties of the products (5).

In the agri-food sector, the use of bacteriophages is increasingly recognized by regulatory authorities. In the United States, phage-based preparations have received the Generally Recognized as Safe (GRAS) status from the Food and Drug Administration (FDA), whereas the European regulatory framework is more restrictive, as the European Food Safety Authority (EFSA) has excluded phages from its Qualified Presumption of Safety (QPS) list, requiring a case-by-case safety assessment (6, 7). Several products have nevertheless been approved in multiple countries, including those in the European Union, Switzerland, Israel, Canada, the United States, Australia, and New Zealand, all composed exclusively of strictly lytic phages that ensure safety and predictable antibacterial activity (8, 9). In contrast, temperate phages are not accepted for direct biocontrol due to their lysogenic potential, although they remain valuable research models and sources of bioactive enzymes such as endolysins (8, 10, 11). The greater applicability of lytic phages has created a research bias in their favor, leaving temperate phages comparatively underexplored. Yet, advances in sequencing and synthetic biology now provide new opportunities to investigate temperate phages, whose biological functions and ecological roles remain highly relevant (12–14).

Regarding ACB-specific phages, publicly available information is scarce. In 1977, Sakaki et al. characterized a lipid-containing phage isolated from a hot spring that targets an *Alicyclobacillus acidocaldarius* (formerly *Bacillus acidocaldarius*) strain. This earlier investigation evaluated the phage’s growth, chemical composition, and stability under different environmental conditions (e.g., different pH levels, temperatures, and solvents). However, the temperate or lytic nature of the phage was not assessed (15). Recently, *Alicyclobacillus* phage KKP 3916 was reported to target different ACB strains from three distinct species, *A. acidocaldarius*, *A. acidoterrestris,* and *Alicyclobacillus fastidiosus* (16). This phage carried several genetic elements indicating a temperate lifestyle, making it not ideal for food biocontrol for the reasons explained above, and its biological role was not further investigated. With only two ACB-specific phages described to date, further studies are essential not only to explore strategies for mitigating spoilage and meeting clean-label demands but also to uncover fundamental biological aspects of these underexplored thermoacidophilic bacteria relevant to food production.

In preliminary screening, *A. acidoterrestris* DSM 3922^T^ and a food isolate, *A. acidoterrestris* MMB007, were evaluated as hosts for phage isolation from environmental samples, including soil and fruit-processing water samples. A phage plaque was isolated using a vineyard soil sample and *A. acidoterrestris* DSM 3922^T^ as host. During purification, the isolated phage lost infectivity toward this strain while remaining active against *A. acidoterrestris* MMB007. This study aimed to characterize the isolated phage, *Alicyclobacillus* phage MMB025, in terms of morphology, genome features, host range, and stability, and to predict its lifestyle. The analyses revealed that MMB025 represents the first infectious phage with genomic identity to a prophage described in the genus ACB, integrated within the sporulation sigma factor gene *sigK*.

## RESULTS AND DISCUSSION

### Isolation, morphology, and host range of *Alicyclobacillus* phage MMB025

In this study, the isolated phage was named *Alicyclobacillus* phage MMB025 (hereafter designated phage MMB025), considering the informal guidelines provided by Adriaenssens and Rodney Brister (17). The phage MMB025 was initially detected following enrichment of a vineyard soil sample with *A. acidoterrestris* DSM 3922^T^. However, subsequent attempts to propagate the phage using the type strain for purification were unsuccessful, despite successful and reproducible infection of the food isolate *A. acidoterrestris* MMB007. The incapacity to infect DSM 3922^T^ was observed after the initial plaque isolation step. Although the conditions allowing initial phage plaque formation remain elusive, subsequent analyses, as detailed below, are consistent with *A. acidoterrestris* DSM 3922^T^ being capable of displaying superinfection immunity to phage MMB025. The potential temperate nature of phage MMB025 is also examined in detail in later sections.

**Fig 1:**
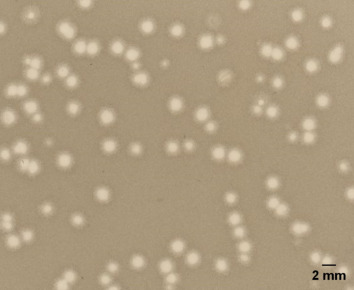
Plaque morphology of *Alicyclobacillus* phage MMB025 on an *A. acidoterrestris* MMB007 lawn. Representative double-layer agar plate showing clear plaques with turbid halos formed by phage MMB025 on *A. acidoterrestris* MMB007 (BAT soft agar, 0.5% [wt/vol]) after 24 h at 45°C. Mean plaque diameter was 1.2 mm ± 0.3 mm (means from *n* = 20 plaques measured). Scale bar, 2 mm; generated in ImageJ (v1.54p).

**Fig 2:**
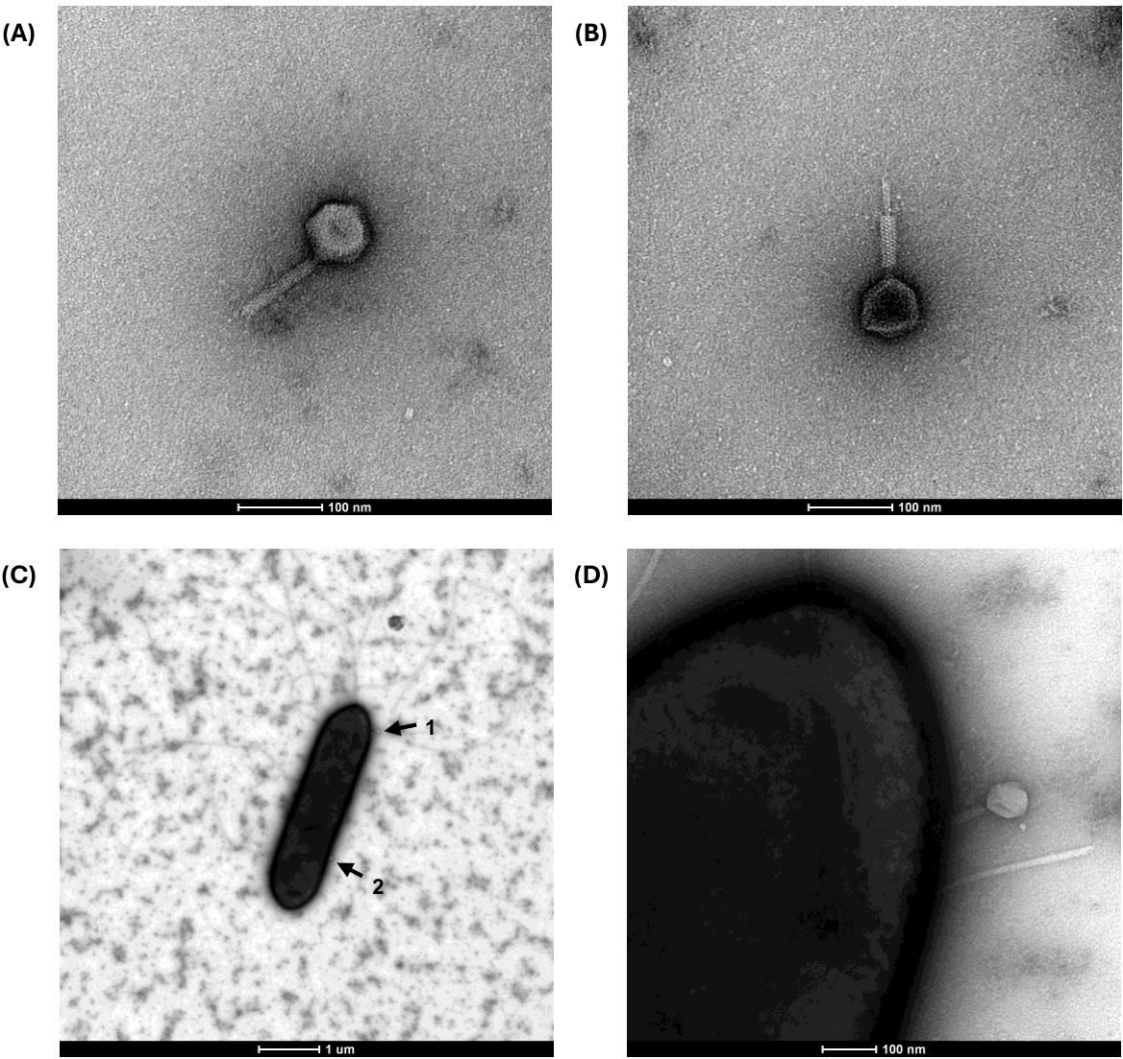
Virion morphology and host attachment of *Alicyclobacillus* phage MMB025 visualized by TEM. (**A**) Negatively stained virion with uncontracted tail; (**B**) Negatively stained virion with contracted tail; (**C**) *A. acidoterrestris* MMB007 showing multiple phage MMB025 virions attached to the cell surface (black arrows); (**D**) Magnified view of panel C (region marked by arrow 1). Samples were fixed (2% formaldehyde), stained with 2% uranyl acetate, and imaged on a FEI Tecnai G2 Spirit BioTWIN at 120 kV; images captured with an Olympus-SIS Veleta CCD. Scale bars: A, B, D = 100 nm; C = 1 µm. Capsid diameter: ~65 nm; contractile tail: ~100 nm × 19 nm.

The isolated phage was characterized based on its morphological features. The phage MMB025 forms clear plaques (diameter 1.2 mm ± 0.3 mm) surrounded by turbid halo zones on an *A. acidoterrestris* MMB007 lawn (Fig. 1). The analysis of the phage MMB025 by TEM revealed that it consists of an isometric capsid (diameter ~65 nm) and a contractile tail (~100 nm × 19 nm) (Fig. 2; Fig. S1). These features suggest that the phage exhibits a contractile-tailed morphotype within the *Caudoviricetes* class, which encompasses a class of tailed phages whose hosts are bacteria and archaea, accounting for more than 90% of the total characterized phages (18, 19).

To assess the host range of phage MMB025, its lytic activity was tested against different isolates within the ACB genus, including both commercially obtained type strains and isolates recovered from food matrices (Table 1). To further investigate the specificity of this lytic activity beyond the ACB genus, the phage MMB025 was tested against bacteria from other genera. These included closely related bacteria such as *Bacillus* spp. and thermophilic bacteria like *Geobacillus stearothermophilus*, as well as hygiene indicator bacteria commonly monitored in the food industry, specifically *Staphylococcus aureus* and *Escherichia coli* (Table 1). These assays revealed the specificity of phage MMB025 for ACB isolates, as no lytic activity was observed against bacteria from different genera (Table 1). The phage MMB025 exhibited a narrow host range, producing plaques on a limited subset of the tested ACB isolates. Specifically, it inhibited the growth of isolates belonging to only two species: *A. acidoterrestris* (7 of 17 isolates), the species most closely correlated with food spoilage events, and *A. acidocaldarius* (1 of 2 isolates), with no plaques observed on other species or genera tested. Among the isolates exhibiting lysis, two distinct plaque morphologies were observed: (i) a complete-lysis phenotype, characterized by clear plaques (“++” in Table 1), and (ii) a medium-lysis phenotype, exhibiting turbid plaques (“+” in Table 1). Representative examples are shown in Fig. S2.

**TABLE 1:** Host range of *Alicyclobacillus* phage MMB025 on tested bacterial strains^*a*^

Bacterial species	Phage lytic activity (no. of isolates with determined phenotype/total of isolates tested)		
	−	+	++
*Alicyclobacillus acidiphilus*	1/1		
*Alicyclobacillus acidocaldarius*	1/2	1/2	
*Alicyclobacillus acidoterrestris*	9/17	1/17	6/17
*Alicyclobacillus contaminans*	1/1		
*Alicyclobacillus cycloheptanicus*	2/2		
*Alicyclobacillus fastidiosus*	1/1		
*Alicyclobacillus fructus*	1/1		
*Alicyclobacillus herbarius*	1/1		
*Alicyclobacillus hesperidum*	1/1		
*Alicyclobacillus kakegawensis*	1/1		
*Alicyclobacillus macrosporangiidus*	1/1		
*Alicyclobacillus mali*	1/1		
*Alicyclobacillus pomorum*	1/1		
*Alicyclobacillus sacchari*	1/1		
*Alicyclobacillus suci*	1/1		
*Alicyclobacillus tengchongensis*	1/1		
*Alicyclobacillus vulcanalis*	1/1		
*Bacillus* spp.	2/2		
*Escherichia coli*	1/1		
*Geobacillus stearothermophilus*	1/1		
*Staphylococcus aureus*	1/1		

^*a*^ Plaque phenotypes are indicated as follows: ++, clear plaques; +, turbid plaques; −, no plaques detected. Gray-shaded cells indicate tested conditions with no isolates matching the indicated plaque phenotype. Results represent two independent biological replicates. Detailed strain information is provided in Table S1.

### Adsorption profile and interaction with host cells

**Fig 3:**
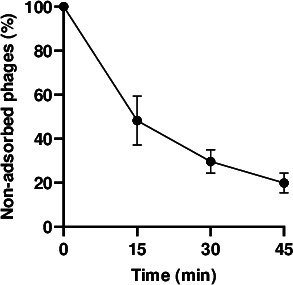
Adsorption profile of *Alicyclobacillus* phage MMB025 to the *A. acidoterrestris* MMB007 host. Fraction of non-adsorbed phages over time after infecting 10^6^ CFU/mL of the host at an MOI of ~0.1 (45°C in BAT broth). The results presented are mean values from three independent experiments, with standard deviations shown by error bars. Non-adsorbed PFU at t = 0 min was set to 100% for normalization.

A critical factor influencing the efficiency of phage infection is the rate at which virions adsorb to their bacterial hosts. A high adsorption rate enables more rapid and more effective bacterial elimination (20, 21). The rate at which phages attach to bacterial cells can be influenced by several factors, including multiplicity of infection (MOI), temperature, pH, and any elements affecting host receptor availability and phage stability. In this study, the adsorption of phage MMB025 to *A. acidoterrestris* MMB007 was evaluated by infecting the host at low cell densities, ~10^6^ colony-forming units per milliliter (CFU/mL), which already represents a high contamination level in food products. An MOI of approximately 0.1 was selected, and the mixture was incubated at 45°C. This setup was designed to simulate scenarios unfavorable for phage-host encounter and interaction, while fully supporting the optimal temperature for bacterial host growth, therefore providing a conservative estimate of adsorption performance under suboptimal conditions. The percentage of non-adsorbed phages was then determined at different time points. Under these conditions, phage MMB025 adsorbs to host cells at a slow rate, with approximately 80% of the phages attaching to bacteria only after 45 min of infection (Fig. 3). However, adsorption efficiency could be higher in conditions that promote cell–phage interactions, such as in the presence of higher concentrations of both bacteria and virions.

### *Alicyclobacillus* phage MMB025 stability under acidic and heat stress

**Fig 4:**
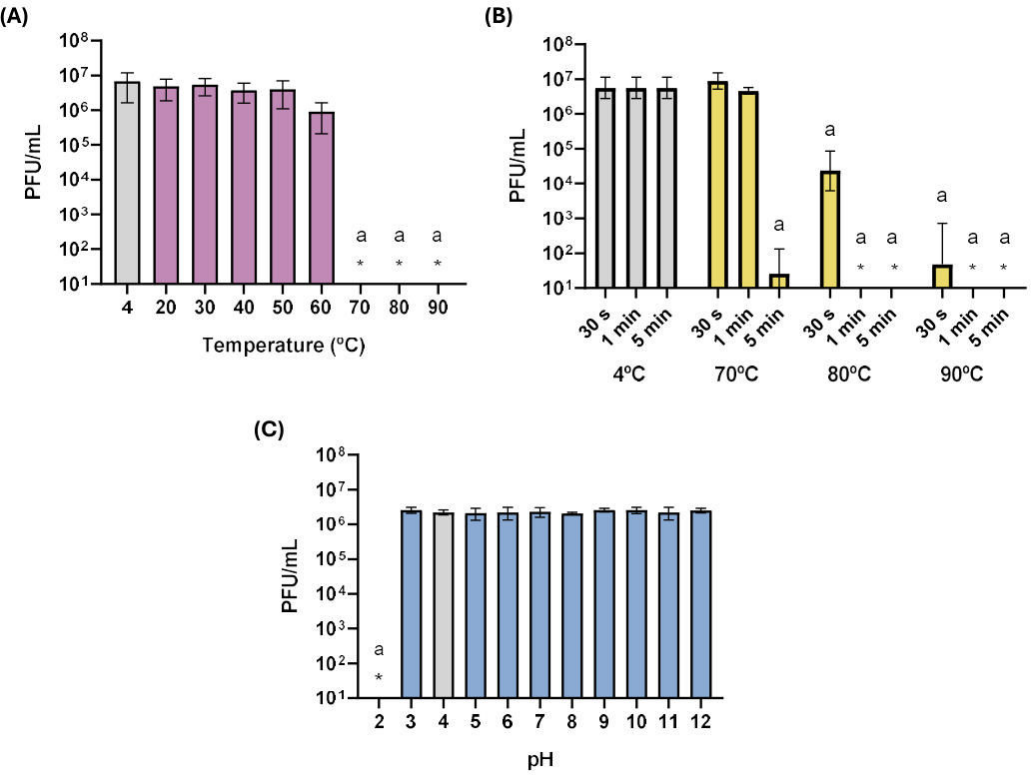
Thermal and pH stability of *Alicyclobacillus* phage MMB025. Stability assessed by titrating phage suspensions following exposure to different conditions: (**A**) Phage titers after 1 h at the indicated temperatures; (**B**) Phage titers after short periods (30 s, 1 min, and 5 min) at the highest temperatures (with 4°C used as control); and (**C**) Phage titers after 1 h at the indicated pH values. Results are presented as the mean of three independent experiments, with error bars indicating standard deviations. “*” indicates values below the LOD of the method. An ordinary one-way ANOVA for (**A**) and (**C**) or two-way ANOVA for (**B**) was performed for each condition compared to the control (gray bars), which represent the storage conditions of the phage stock solution used in the assay. Conditions with *p*-values < 0.05 are marked with “a.”

The assessment of phage stability under various environmental conditions is crucial not only for predicting phage behavior in the environment but also for evaluating potential industrial applications. *A. acidoterrestris* are thermoacidophilic bacteria that grow within a pH range of approximately 2.0–6.0 and at temperatures between 20°C and 60°C, with an optimum around 45°C. These conditions are representative of its natural habitats and of fruit-processing environments prior to pasteurization, where acidic pH values predominate. Therefore, the stability of phage MMB025 was evaluated across temperatures and pH ranges relevant to these niches (Fig. 4). The phage maintained its titer when subjected to a wide range of temperatures for 1 h, withstanding up to 60°C, and experiencing at most about a 1 log reduction at this temperature (Fig. 4A). For the higher temperatures of 70°C, 80°C, and 90°C, reduced exposure times were tested, since 1-h exposures resulted in values below the limit of detection (LOD) of the method (Fig. 4B). The phage MMB025 demonstrated good stability at 70°C for 30 s and 1 min, with some virions still resisting a 5-min exposure, albeit with higher variations and a significant reduction of at least 5 log (Fig. 4B). At 80°C and 90°C, the phage titer significantly decreased compared to the positive control, maintained at 4°C, even with exposure times as brief as 30 s (Fig. 4B). When exposed to a wide range of pH levels, phage MMB025 retained its activity at pH values of 3 and above (Fig. 4C). The stability of phage MMB025 under a wide spectrum of pH and up to 60°C indicates robustness under acidic environments and moderately thermic conditions relevant to ACB niches, highlighting that the phage MMB025 remains stable under conditions commonly encountered during fruit-processing steps prior to pasteurization.

### Genomic architecture and evidence for a headful packaging mechanism

**Fig 5:**
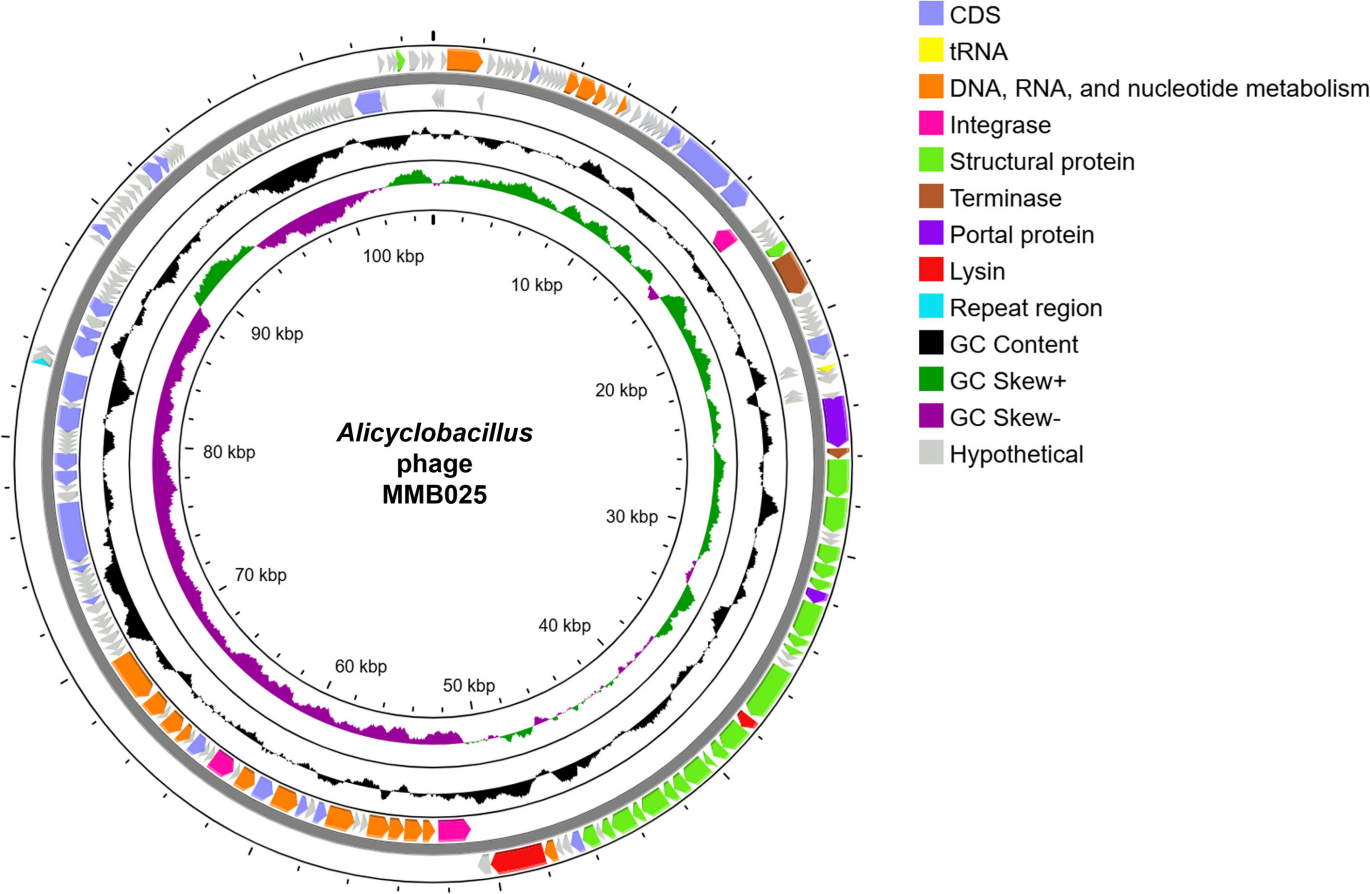
Circular genome map of *Alicyclobacillus* phage MMB025 (105,243 bp; 43.3% GC). From outside inward: coding sequence (CDS) regions identified on the forward strand, CDS regions identified on the reverse strand, GC content, and GC skew. Genetic features and CDS functional groups are highlighted according to the color code. The map is oriented at the predicted pac site (PhageTerm call). Figure adapted from Proksee.

The hybrid sequencing of phage MMB025 revealed its genome consists of a 105,243 bp linear dsDNA molecule (43.3% GC). PhageTerm analysis of the assembly supported a headful (pac) packaging mechanism, with read-coverage features at a predicted pac site corroborated by both short- and long-mapping (Fig. S3). Annotation tools (22–26) predicted a total of 214 CDS, including 73 with putative functions and 141 CDS related to hypothetical proteins (Fig. 5; Table S2). Among the genes with assigned functions, roles associated with DNA replication and transcription regulation (e.g., DNA polymerases), genome packaging (e.g., small and large terminase subunits), virion structure (e.g., head, tail, and baseplate-related proteins), lysis (e.g., putative endolysins), and lysogeny (e.g., putative integrases) were identified (27–30). Three genes encoding putative integrases were identified, suggesting that phage MMB025 has a temperate nature, as integrases mediate the integration of the phage DNA into the host genome (31). Also, no antibiotic resistance genes, virulence factors, or anti-phage systems were detected (32–35). The phage genome map is shown in Fig. 5, highlighting the annotated functional regions, including CDSs related to DNA/RNA/nucleotide metabolism, integrases, structural proteins, terminase subunits, portal-associated proteins, and lysis functions (36).

**Fig 6:**
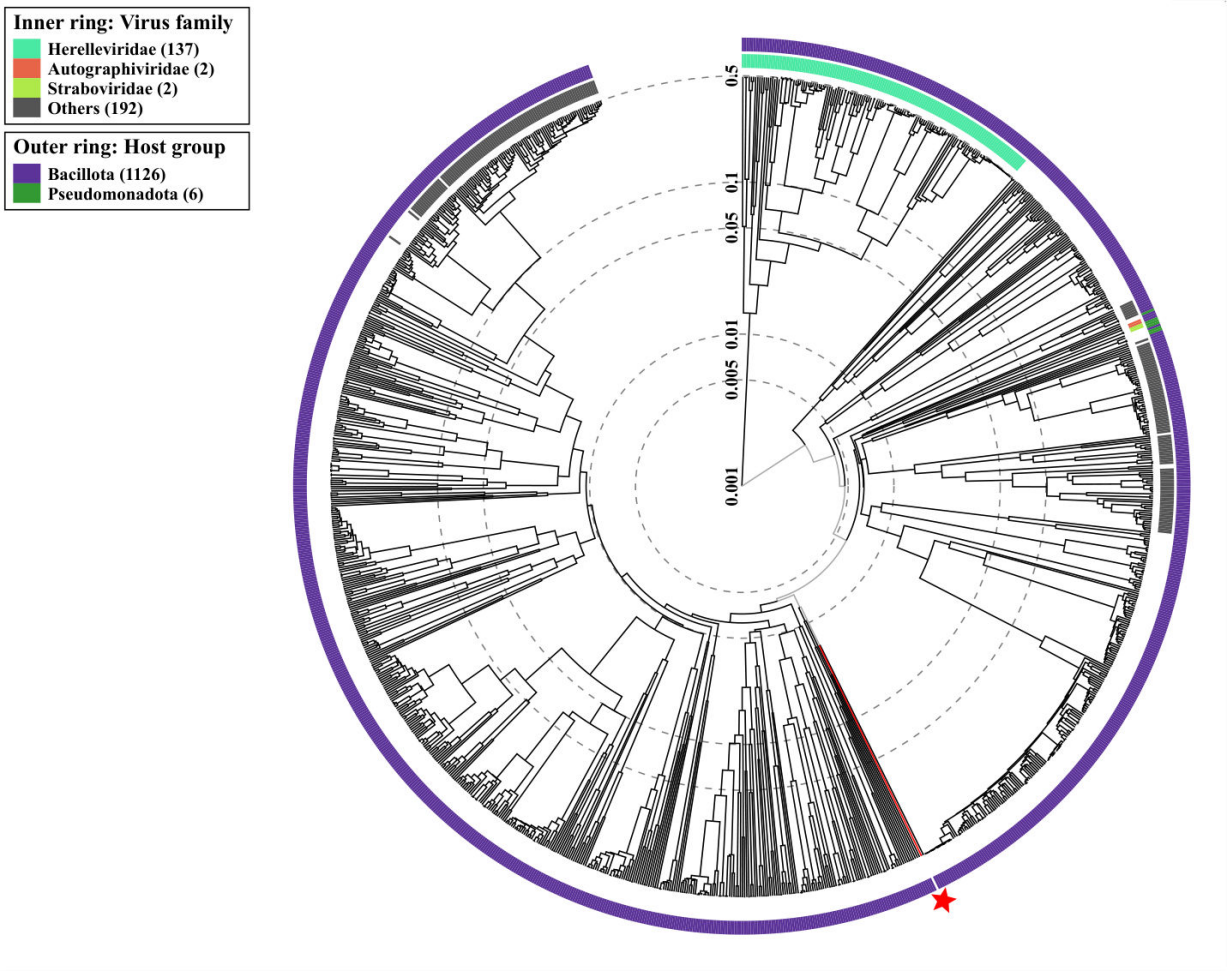
Proteomic tree positioning of *Alicyclobacillus* phage MMB025 within *Caudoviricetes*. The displayed tree was generated using the ViPTree server. It includes the phage MMB025 sequence (red star), all available sequences of phages targeting bacteria from the *Bacillota* phylum, and six phage sequences targeting bacteria from the distant *Pseudomonadota* phylum. Sequence and taxonomic data are based on the Virus-Host DB. It was calculated by BIONJ based on the genomic distance matrix and rooted at the midpoint. The tree is presented in a circular view with log-scaled branch lengths. The outer rings indicate the host phylum, and the inner rings denote the phage family.

Additionally, the phage MMB025 taxonomy was predicted based on its genomic sequence (37). The analysis of phage MMB025, alongside phages infecting closely related hosts within the phylum *Bacillota*, determined that it belongs to the *Caudoviricetes* class (Fig. 6), although its specific family could not be identified. The results from VipTree indicate that this phage is well-separated from the classified *Herelleviridae*, *Autographiviridae*, and *Straboviridae* families and is more closely related to phages from unclassified families. A direct comparison between the phage MMB025 and the previously reported *Alicyclobacillus* phage KKP 3916, conducted through MAFFT alignment, revealed only 34.3% identity, suggesting they are not closely related, despite both having ACB bacteria as host (16). A similar comparison with the phage described by Sakaki et al. (1977) was not possible because its genome has never been sequenced, and the phage is no longer available through any culture collection (15). Additionally, no closely related phages were identified using the taxMyPhage classification tool, underscoring the limited genomic representation of ACB-related phages and suggesting that the phage MMB025 represents a distinct lineage within the *Caudoviricetes*, consistent with the possibility that it constitutes a novel genus (38).

### Phage lifestyle and integration within the sporulation gene *sigK*

The lifestyle of a phage is a determining factor in its application as an antibacterial agent. While strictly lytic phages are suitable for direct application into food products due to their immediate bactericidal activity, temperate phages pose challenges, as they can integrate into bacterial genomes and potentially promote phage resistance in target bacteria through superinfection immunity and exclusion mechanisms (39–41). Therefore, further analyses were performed to try to predict the phage MMB025 lifestyle.

**Fig 7:**
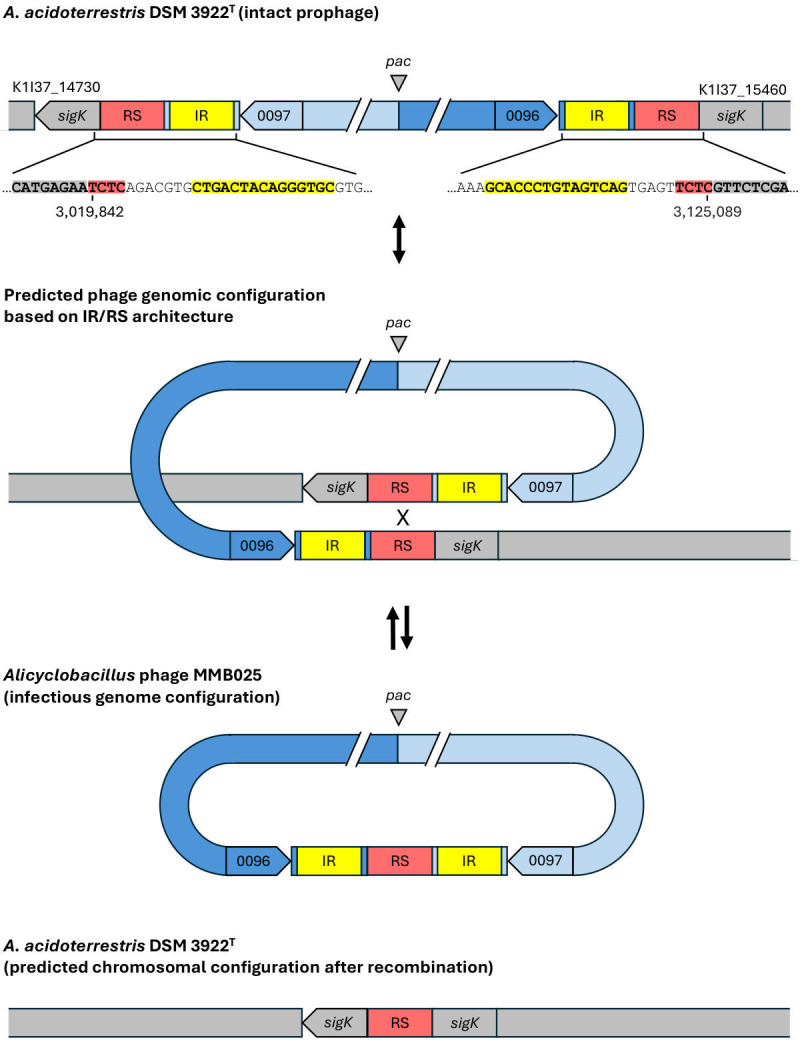
Genomic reconstruction of site-specific integration of *Alicyclobacillus* phage MMB025 at the *sigK* locus of *A. acidoterrestris* DSM 3922^T^. Schematic (not to scale) of prophage integration disrupting *sigK*, with the bacterial genome sequence shown in gray, while the phage MMB025 is depicted in blue. Regions identified as inverted repeats (IR) and recombination sites (RS) are highlighted in yellow and red, respectively. Top: native genomic organization of the bacterial locus, containing the *sigK* gene interrupted by the phage genome containing the pac site, and the recombination region featuring IR and RS, with flanking ORFs marked by arrows; Middle and bottom: predicted configuration of the phage genome and chromosomal locus based on conserved IR/RS architecture and comparative genomic alignment. Junctions and orientation were supported by read mapping and MAUVE alignment; the complete genome sequence of phage MMB025 (GenBank accession number PV686781) and the complete genome of *A. acidoterrestris* DSM 3922^T^ (GenBank accession number CP080467) were used; see Fig. S4 for alignments and coordinates.

As mentioned earlier, the analysis of the phage MMB025 genome revealed genetic elements pointing to a temperate nature. Strikingly, BLASTN and MAUVE analysis revealed that the phage MMB025 genome assembled in this study mapped with 99.99% identity to a chromosomal region of the type strain *A. acidoterrestris* DSM 3922^T^, with a rearrangement pattern consistent with prophage integration (Fig. S4). This rearrangement is expected if the recombination site for viral DNA integration in the bacterial chromosome lies roughly in the middle of the phage genome. These findings strongly suggest that phage MMB025 corresponds to an infectious form of a prophage of *A. acidoterrestris* DSM 3922^T^, which could explain the incapacity of the phage to infect this strain due to superinfection immunity. In addition, the average GC content of the complete genome of *A. acidoterrestris* DSM 3922^T^ is approximately 52.3%, whereas phage MMB025 exhibits a GC content of 43.3%, indicating that the prophage region corresponds to a distinct genomic feature that may reflect its evolutionary divergence from the host (42, 43). Interestingly, precise mapping of the bacterial-prophage DNA junctions revealed that the phage is integrated within an ORF encoding a sigma factor (Fig. 7). When reconstructed in its uninterrupted form, this ORF shows high sequence identity (>93%) to the *sigK* gene found in other ACB species, such as *A. suci* and *A. fructus* (44). The integrated phage genome is flanked by inverted repeat (IR) sequences, each followed by a conserved 4-nucleotide motif (TCTC), which likely functions as the core recombination site (RS, Fig. 7). This recombination site, located at the position 50,867 bp–50,870 bp of the phage genome sequence, is immediately upstream of a gene encoding a putative integrase carrying a conserved domain of the SpoIVCA family (OUHLZNSR_CDS0097). This genomic architecture—comprising IRs, a short RS, and a proximal site-specific recombinase—is characteristic of site-specific recombination modules commonly found in temperate phages and mobile genetic elements. Although the TCTC motif is unusually short for a recognized recombination site, similar small motifs have been reported in mobile elements of other *Bacillota*. A well-characterized example is the *sigK*-intervening (*skin*) element in *B. subtilis*, a 48 kb prophage-like region that interrupts the *sigK* gene with a core RS composed of only five nucleotides (45–47). Unlike typical prophages, the *skin* element does not produce phage particles but plays a regulatory role in sporulation. It is precisely excised by the SpoIVCA recombinase during the late stages of sporulation, restoring the functional *sigK* gene and enabling the transcription of late sporulation genes in the mother cell (47). This mechanism has also been described in other spore-forming *Bacillota*, including *Clostridium difficile* and *Clostridium perfringens* (48). These data suggest a similar sporulation-associated regulatory pathway mediated by prophage excision in *A. acidoterrestris*. The integration of the phage MMB025 within the *sigK* locus, along with the presence of recombination-associated features, suggests a functional parallel to the *skin* element. However, unlike the *skin* elements of *B. subtilis, C. difficile,* and *C. tetani*, which do not produce phage particles, the *A. acidoterrestris* DSM 3922^T^ prophage appears to retain the ability to form infectious phage particles, similarly to phage ϕS63 that also integrates in the *sigK* gene of *Clostridium perfringens* (49).

Online bioinformatic tools were used to verify both prophage regions within the genome of *A. acidoterrestris* DSM 3922^T^ (PHASTEST) and the temperate nature of phage MMB025 (BACPHLIP, PhageScope, and PhageAI tools) (50–53). A 35,615 bp region, located between the positions 3,082,161 and 3,117,776 of the *A. acidoterrestris* DSM 3922^T^ genome, was predicted by PHASTEST as phage-related, with a confidence score of 80. This region was entirely covered by part of the assembled phage MMB025 genome (positions 7,943 to 43,559). However, the complete genome of phage MMB025 (105,243 bp) extends substantially beyond the boundaries predicted by PHASTEST. This discrepancy likely reflects limitations of similarity-based prophage detection tools, which may underestimate the full prophage boundaries when analyzing novel or divergent temperate phages. Direct mapping of the experimentally sequenced MMB025 genome to the host chromosome revealed near-complete nucleotide identity (99.99%) across the entire 105 kb region, supporting that the full-length sequence corresponds to an integrated prophage locus. Additionally, the sequence-based tools used to predict the lifestyle of phage MMB025 revealed inconsistencies. BACPHLIP suggested that the phage has a 100% probability of being a temperate phage, while PhageScope indicated a 100% virulent nature for the phage, and PhageAI predicted a 77.02% probability of the phage being virulent. These conflicting predictions highlight the limitations of sequence-only classifiers and emphasize the need for integrated bioinformatic approaches and experimental validation, particularly for non-model taxa.

The potential functional consequences of *sigK* interruption in *A. acidoterrestris* DSM 3922^T^, including any effects on sporulation regulation, remain to be experimentally determined. Targeted experiments should aim to determine whether the excision of the phage occurs during late-stage sporulation, thereby restoring a functional *sigK* gene. More importantly, it is crucial to characterize the conditions under which phage MMB025 switches between lysogenic and lytic cycles.

### Predicted lytic enzymes and lysin candidates from the MMB025 genome

Considering its genomic features and origin, phage MMB025 was explored as a source of candidate lysins and other enzymes for future testing. Genomic analysis using Pharokka and phold annotated two genes coding for putative products with endolysin features: OUHLZNSR_CDS0078 (37,614 bp–38,147 bp; 534 bp; 177 aa) and OUHLZNSR_CDS0095 (47,982 bp–50,243 bp; 2,262 bp; 753 aa). Endolysins are enzymes that mediate bacterial cell-wall disruption through peptidoglycan degradation at the end of the lytic cycle (54). HHpred was applied to assess the likelihood of each protein possessing catalytic domains characteristic of muralytic activity. OUHLZNSR_CDS0078 was identified as a LysM domain-containing protein showing higher homology to tail tube initiator proteins (55). Although the LysM domain is involved in peptidoglycan recognition, no catalytic domains associated with peptidoglycan degradation were detected, suggesting that this protein is more likely to play a structural rather than a lytic role (56). In contrast, HHpred identified two domains commonly associated with bacterial cell wall degradation in OUHLZNSR_CDS0095, a peptidase and a muramidase domain, suggesting a likely function in peptidoglycan degradation (57).

In addition to these putative endolysins, genes encoding proteins responsible for fatty acid degradation may also be of interest for eliminating ACB bacteria. The ω-alicyclic fatty acids are known to be related to ACB resistance to acidic pH and high temperatures and constitute the major component of ACB cellular membranes (58, 59). In this study, one CDS with predicted enoyl-CoA hydratase/carnitine racemase-like function (OUHLZNSR_CDS0091) was identified in the phage MMB025 genome. This dual annotation reflects sequence homology to and between both enzyme families, typically associated with fatty acid metabolism and degradation (60, 61). Further structure and function prediction using HHpred revealed that the 168-amino acid sequence shares similarities with cysteine peptidase and phospholipase domains. While this complicates the functional assignment, it suggests a potential role in membrane remodeling and disruption.

The identification of these enzymes highlights the potential of temperate phages like MMB025 as reservoirs of biotechnologically relevant enzymes, even when not directly applicable as biocontrol agents in food matrices.

### Concluding remarks

This study offers the first detailed characterization of *Alicyclobacillus* phage MMB025, an infectious phage with genomic identity to a prophage from the genus ACB. Phage MMB025 exhibits a contractile-tailed morphology and a narrow host range while maintaining remarkable stability across acidic and moderate thermal conditions. Hybrid sequencing revealed a 105 kb genome encoding structural, replication, packaging, lysis, and lysogeny modules, with no detectable antibiotic resistance or virulence genes. MMB025 resides as a prophage in the *A. acidoterrestris* DSM 3922^T^ genome integrated within the sporulation sigma factor gene *sigK*, suggesting a sporulation-associated regulatory role analogous to the *B. subtilis skin* element. Among the predicted proteins, a multi-domain lysin and other enzymes were identified as promising candidates for future functional studies.

Collectively, these findings expand our knowledge on ACB phages, uncover the first evidence of prophage-sporulation interactions in this genus, and highlight temperate phages as overlooked sources of enzymes with potential relevance to food spoilage mitigation. Further molecular and biochemical analyses will clarify the mechanisms governing induction, excision, and lysin activity, advancing both fundamental and applied understanding of thermoacidophilic spore-forming bacteria.

## MATERIALS AND METHODS

### Bacteria and growth conditions

Immediate sources of all bacterial strains are provided in Table 2; culture collection strains were obtained directly from DSMZ, ATCC, BGSC, or CECT, and food isolates were obtained from the Microbiology and Molecular Biology Laboratory (iBET, PT) and the Microbial Food Safety and Spoilage Laboratory (Cornell University, USA).

**TABLE 2:** Bacterial strains used in this study

Bacterial strain	Origin	Reference
Culture collection strains		
*A. acidiphilus* DSM 14558^T^	Acidic beverage	DSMZ (Germany)
*A. acidocaldarius* DSM 446^T^	Acid hot spring	DSMZ (Germany)
*A. acidoterrestris* DSM 3922^T^	Soil	DSMZ (Germany)
*A. contaminans* DSM 17975^T^^*a*^	Soil	DSMZ (Germany)
*A. cycloheptanicus* DSM 4006^T^	Soil	DSMZ (Germany)
*A. cycloheptanicus* ATCC 49029^*a*^	Soil	ATCC (USA)
*A. fastidiosus* DSM 17978^T^	Apple juice	DSMZ (Germany)
*A. fructus* DSM 112018^T^^*a*^	Mixed fruit juice concentrate	DSMZ (Germany)
*A. herbarius* DSM 13609^T^^*a*^	Dry flower of *Hibiscus*	DSMZ (Germany)
*A. hesperidum* DSM 11985^T^	Solfataric soils	DSMZ (Germany)
*A. kakegawensis* DSM 17979^T^^*a*^	Soil	DSMZ (Germany)
*A. macrosporangiidus* DSM 17980^T^^*a*^	Soil	DSMZ (Germany)
*A. mali* DSM 112016^T^^*a*^	Pasteurized pear juice	DSMZ (Germany)
*A. pomorum* DSM 14955^T^^*a*^	Mixed fruit juice	DSMZ (Germany)
*A. sacchari* DSM 17974^T^^*a*^	Liquid sugar	DSMZ (Germany)
*A. suci* DSM 112017^T^^*a*^	Apple juice	DSMZ (Germany)
*A. tengchongensis* ATCC BAA2134^T^^*a*^	The soil of a hot spring soil	ATCC (USA)
*A. vulcanalis* ATCC BAA915^T^^*a*^	Hot spring pool water	ATCC (USA)
*B. subtilis* CECT 356	Not specified by the culture collection	CECT (Spain)
*B. cereus* BGSC 6A1	Not specified by the culture collection	BGSC (USA)
*E. coli* ATCC 25922	Clinical isolate	ATCC (USA)
*G. stearothermophilus* DSM 22^T^	Deteriorated canned food	DSMZ (Germany)
*S. aureus* ATCC 6538	Clinical isolate, human lesion	ATCC (USA)
In-house culture collection strains		
*A. acidocaldarius* MMB020	Tomato paste	This study
*A. acidoterrestris* MMB001	Passion fruit concentrate	This study
*A. acidoterrestris* MMB002	Peach juice	This study
*A. acidoterrestris* MMB003	Peach juice	This study
*A. acidoterrestris* MMB004	Fruit mix concentrate	This study
*A. acidoterrestris* MMB005	Pear juice	This study
*A. acidoterrestris* MMB006	Peach juice	This study
*A. acidoterrestris* MMB007	Tangerine concentrate	This study
*A. acidoterrestris* MMB008	Sugar	This study
*A. acidoterrestris* MMB009	Tangerine concentrate	This study
*A. acidoterrestris* MMB010	Mango and orange juice	This study
*A. acidoterrestris* MMB011	Peach juice	This study
*A. acidoterrestris* MMB012	Peach juice	This study
*A. acidoterrestris* MMB013	Peach juice	This study
*A. acidoterrestris* MMB021	Peach juice	This study
*A. acidoterrestris* FSL R14-0029^*a*^	Apple juice	(62)
*A. acidoterrestris* FSL R14-0046^*a*^	Apple juice	(62)
*A. suci* FSL-R14-0023^*a*^	Apple juice	(62)
*A. suci* FSL-R14-0051^*a*^	Apple juice	(62)

^*a*^ Culture collection strains and food industry isolates were tested in collaboration with Dr. Abby Snyder and Dr. Katerina Roth from the Microbial Food Safety and Spoilage Lab, Cornell University, Ithaca, NY, USA.

ACB strains, both from culture collections and food isolates, were routinely grown in BAT agar (BAT broth [Scharlau, Spain] supplemented with 1.5% [vol/vol] agar [VWR, USA]) and incubated at 45°C. To prepare liquid cultures, fresh colonies were grown in BAT broth at 45°C with shaking at 180 rpm in a New Brunswick Innova 42R incubator (Germany). Before use, both BAT agar and BAT broth were adjusted to a pH of 4.0 using a 1 M HCl solution.

*B. subtilis* CECT 356, *B. cereus* BGSC 6A1*, G. stearothermophilus* DSM 22, *S. aureus* ATCC 6538, and *E. coli* ATCC 25922 were also included in this study to evaluate phage host specificity beyond the ACB genus. They were all grown in Luria-Bertani broth (LB), prepared by adding 10 g/L tryptone (MilliporeSigma, Switzerland), 5 g/L yeast extract (Thermo Scientific, USA), and 10 g/L NaCl (VWR Chemicals, USA), or in Luria-Bertani agar (LA) if supplemented with 15 g/L agar. Before use, both LB and LA were adjusted to pH 7.0. Incubation was performed at 37°C, except for *G. stearothermophilus*, which was incubated at 55°C, with shaking at 180 rpm in a New Brunswick Innova 42R for liquid cultures.

Whenever required, bacterial cultures were standardized to approximately 10^6^ CFU/mL. For this, preliminary growth curve analyses were performed for each strain to establish the correlation between optical density at 600 nm (OD_600_) and viable counts (CFU/mL) during exponential growth. Once the cells reached the mid-exponential growth phase, bacterial cultures were normalized using the predetermined absorbance (OD_600_) and, if required, serially diluted to achieve an inoculum with the desired concentration.

### Phage isolation and purification

Bacterial concentrations were expressed as colony-forming units per milliliter (CFU/mL) and phage concentrations as plaque-forming units per milliliter (PFU/mL), determined by standard spread plating and double-layer agar methods, respectively.

A sample of vineyard soil collected from Quinta do Marquês in Oeiras, Portugal, was processed for the isolation of phages. To achieve this, 10 mL of BAT broth was added to 5 g of soil and homogenized by vortexing. After 10 min of sedimentation at room temperature (22°C), the upper phase was filtered using a 0.45 µm syringe PES filter. The supernatant was tested against *A. acidoterrestris* DSM 3922^T^ and *A. acidoterrestris* MMB007 using an overlay agar assay. For this, fresh liquid cultures of each isolate were grown and used to inoculate 5 mL of BAT soft agar (BAT broth supplemented with 0.5% [wt/vol] agar) with approximately 10^6^ CFU/mL. A volume of 500 µL of the filtered supernatant was mixed with the inoculated BAT soft agar, which was poured onto a previously solidified 20 mL BAT agar base. After 24 h of incubation at 45°C, the plates were evaluated for the presence of phage plaques.

The phage initially obtained on a lawn of *A. acidoterrestris* DSM 3922^T^ was purified according to the protocol established by Shymialevich et al. (16), with a few modifications. Briefly, an individual plaque was recovered using a sterile Pasteur pipette and transferred to tubes containing 1 mL of BAT broth, before being incubated at 45°C on a digital heating shaking dry bath (300 rpm) for 24 h to allow the viral particles to diffuse from the phage plaque agar plug into the medium. The resulting phage suspension was filtered using a 0.45 µm PES syringe filter and used for a new overlay agar assay by adding 100 µL of the phage suspension to the soft agar inoculated either with *A. acidoterrestris* DSM 3922^T^ or *A. acidoterrestris* MMB007. Five rounds of single-plaque passage purification steps were performed until plates with homogeneous phage plaque morphology were obtained. The resulting phage suspensions were stored at 4°C.

### Phage titration

Whenever needed, phage suspensions were quantified (titrated) by determining the number of PFU on a lawn of host bacteria (63). To achieve this, phage suspensions were serially diluted in BAT broth, and each dilution was mixed with 100 µL of a fresh culture of the target host *A. acidoterrestris* MMB007 in a 1:1 (vol/vol) ratio. After a 10-min incubation at room temperature (22°C), the mixture was used to inoculate 5 mL of BAT soft agar, which was then poured onto a previously solidified 20 mL of BAT agar base. Plates were incubated at 45°C for 24 h, the phage plaques were enumerated, and the phage titers were expressed as PFU/mL.

### Transmission electron microscopy analysis

The morphological features of the isolated phage were examined using transmission electron microscopy (TEM) imaging. To achieve this, a fresh phage suspension was prepared and fixed with equal volumes of the suspension and a 2% (vol/vol) formaldehyde solution. A sample of target bacteria *A. acidoterrestris* MMB007, which had been exposed to phages at an MOI of 1 for 30 min at 45°C, was also prepared and fixed using the same approach. Samples were kept on ice until analysis. MOI was defined as the ratio of phage particles (PFU) to bacterial cells (CFU) at the time of inoculation.

Sample negative staining and imaging were performed at the Electron Microscopy Facility at the Gulbenkian Institute for Molecular Medicine (GIMM), Oeiras, Portugal (https://gimm.pt). For this, M100 formvar and carbon-coated grids, which had been glow-discharged at 30 mA for 30 s, were used. A volume of 4 µL of each sample was placed on a piece of parafilm, and the grid was positioned on top for adsorption for 2 min. The grid was then washed in 10 drops of distilled water and negatively stained with one drop of 2% (wt/vol) uranyl acetate for 2 min before blotting dry. For imaging, a FEI Tecnai G2 Spirit BioTWIN operating at 120 kV was used, and images were acquired with an Olympus-SIS Veleta CCD Camera. Images were captured at magnifications of 5k, 105k, and 160k, with overviews at 16.5k. ImageJ (v1.54p) was used to determine the dimensions of the virion, measuring the phage head and tail (Fig. S1).

### Adsorption to host cells

To evaluate phage adsorption to *A. acidoterrestris* MMB007, approximately 10^6^ CFU/mL were infected with a phage suspension to achieve an MOI of approximately 0.1 and incubated at 45°C. Every 15 min, 1 mL of culture was collected and centrifuged at 6,000 *g* for 1 min to sediment the cells along with the adsorbed phages. The supernatant was diluted in BAT broth, and the free (non-adsorbed) PFU were quantified as previously described. The number of non-adsorbed phages determined at time 0 min was considered as 100%, and subsequent time points were compared to this sample. Three biological replicates of the phage adsorption assay were conducted.

### Determination of host range

To determine the host range of the phage, agar overlay assays were performed using several bacterial isolates, including multiple species within the ACB genus and others from different genera, such as *Bacillus* spp., *Geobacillus* spp., *Staphylococcus* spp., and *Escherichia* spp. (Table 1). To achieve this, fresh cultures of each bacterial strain or isolate were grown and used to inoculate 5 mL of soft agar (BAT or LB 0.5% [wt/vol] agar) with approximately 10^6^ CFU/mL (64). The inoculated soft agar was poured onto the previously solidified 20 mL of base agar (BAT or LA 1.5% [wt/vol] agar). After drying, 10 µL of serial dilutions of a phage suspension (approximately 10^9^ PFU/mL) were drop-plated on top of the soft agar. After 24 h of incubation, the plates were examined for the presence or absence of phage plaques. Two biological replicates were conducted.

### Thermal and pH stability

Phage stability was evaluated across a broad range of temperatures and pH values to assess its resilience to various environmental conditions. To determine the phage temperature stability, a suspension containing approximately 10^7^ PFU/mL was incubated at varying temperatures—4°C, 20°C, 30°C, 40°C, 50°C, 60°C, 70°C, 80°C, and 90°C—for 1 h. The phage titer was determined after exposure to the distinct temperatures. For conditions where the titer fell below the LOD of 10 PFU/mL, shorter incubation periods were also tested—30 s, 1 min, and 5 min. Similar tests were performed to assess the pH stability of the phage. For this, a suspension with approximately 10^7^ PFU/mL maintained in standard BAT broth was mixed 1:2 (vol/vol) with BAT broth prepared at different pH levels, ranging from 2 to 12, adjusted with 1 M HCl or 1 M NaOH accordingly. After a 1-h incubation, the phage titer was determined. Three biological replicates of these assays were conducted in triplicate.

### DNA extraction and sequencing

High-titer phage lysates were propagated on *A. acidoterrestris* MMB007, the susceptible host used for subsequent DNA extraction. To obtain a phage suspension with the high titer required to extract high-quality DNA (approximately 10^9^ PFU/mL), 20 webbed plates—plates that contain densely packed confluent phage plaques with only a “web” of bacteria left between them—were produced through overlay agar assays. The webbed plates were flooded with 5 mL of BAT broth and incubated for 24 h at 45°C while shaking at 80 rpm in a New Brunswick Innova 42R incubator. The phage suspension was recovered, filtered through a 0.45 µm PES syringe filter, and treated with RNase and DNase at 10 µg/mL each for 30 min at 37°C to digest exogenous nucleic acids, including bacterial. The treated supernatant was then centrifuged for 16 h at 15,000 *g*, at 4°C. The supernatant was discarded, and the pellet was resuspended in 500 µL of BAT broth by shaking at room temperature at 80 rpm in a New Brunswick Innova 42R for 2 h. Phage DNA from the resuspended pellet was extracted using the PureLink RNA/DNA Mini Kit (Thermo Fisher Scientific Inc., USA) according to the manufacturer’s protocol. The extracted DNA was quantified using a NanoDrop One^C^ Microvolume UV-Vis Spectrophotometer (Thermo Fisher Scientific, USA) and sequenced using both Illumina and Oxford Nanopore technologies, described below.

For short-read sequencing, a genomic DNA library was prepared using the NEBNext Ultra II Library Prep Kit for Illumina (New England Biolabs, Ipswich, MA, USA) after DNA fragmentation with a Bioruptor Plus (Diagenode, Belgium) by applying 30 cycles of 30 s ON / 90 s OFF. The library was quantified using the NEBNext Library Quant Kit for Illumina (New England Biolabs, USA), following the manufacturer’s protocol and diluted to 2 nM in 10 mM Tris-HCl. The MiSeq Reagent V2 300 cycle kit (Illumina, San Diego, CA, USA) was used for paired-end sequencing on the MiSeq platform (Illumina, San Diego, CA, USA), with phi X 174 DNA added to the library as an internal control for the reaction.

Long-read sequencing was outsourced to MicrobesNG, Birmingham, United Kingdom (http://www.microbesng.com). Sequencing libraries were generated using SQK-RBK114.96. Sequencing was performed on a GridION (Oxford Nanopore Technologies) using an R10.4.1 flow cell, with base calling model r1041_e82_400bps_sup_variant_v4.3.0.

### Genome assembly and annotation

Before proceeding with phage genome assembly and annotation, preprocessing of short and long reads was carried out using the US Department of Energy Systems Biology Knowledgebase—Kbase (65). Within this open-source software and data platform, the total raw short reads obtained through Illumina sequencing were quality- and length-filtered using Trimmomatic (v0.39) with a sliding window of 5, a quality cut-off of Q30, and a length cut-off of 50 bp (66). The Nextera XT adapters were also removed from the reads. The Oxford Nanopore long reads underwent quality and length filtering using Filtlong (v0.2.1) (https://github.com/rrwick/Filtlong) with a quality cutoff of 90% and a length cutoff of 1,000 bp.

Following this, a *de novo* assembly was performed using SPAdes (v4.1.0) with the basic command option “--metaviral,” which runs the metaviralSPAdes pipeline for virus detection. As input data, a hybrid assembly was conducted using the previously preprocessed short and long reads (67, 68). Phage genome termini and packaging strategy were determined using PhageTerm (v4.1), based on read coverage patterns obtained from both Illumina and Oxford Nanopore data sets (69, 70). A headful (pac-type) packaging mechanism was inferred from a characteristic coverage peak at the predicted pac site, followed by increased read depth consistent with terminal redundancy. The predicted pac site was used to define the genome start position. The assembled genome was manually curated by mapping preprocessed short and long reads back to the assembled contig to resolve ambiguities and confirm base calls using Geneious Prime (v2025.1.2) (https://www.geneious.com). Pharokka (v1.7.5) and phold (v0.2.0) were used in tandem to annotate the curated phage genome (22–26). Finally, the obtained genome was mapped to the *A. acidoterrestris* DSM 3922^T^ genome (42), aligned using the progressiveMauve command, and visualized in Geneious Prime (v2025.1.2) (https://www.geneious.com).

### Genome analysis

HHpred was used to predict protein functional domains from annotated CDSs in the phage genome (71). DefenseFinder (v2.0.0), the Resistance Gene Identifier (v6.0.5), and the Virulence Factor Database (VFDB) (v4.0) were used to investigate anti-phage defense systems, antimicrobial resistance genes, and virulence factors, respectively (32–35). Proksee (v1.3.0) was used for genome visualization (53). The ViPTree server (v4.0) was used to generate a proteomic tree of viral genome sequences based on genome-wide sequence similarities computed by tBLASTx (37, 72). Also, the tool taxMyPhage was employed to search for closely related phages (38). Finally, the *A. acidoterrestris* DSM 3922^T^ genome was analyzed in PHASTEST (v3.0) to predict phage regions, and BACPHLIP (v0.9.6), PhageScope (v1.3), and PhageAI (v1.0.0) were employed to automatically predict phage lifestyle, returning a percentage of virulent/temperate nature (50–53). PHASTEST was also used to predict phage regions in other ACB strains, and Geneious Prime (v2025.1.2) (https://www.geneious.com) was used to map the phage MMB025 sequence to available ACB genomes.

## Data Availability

The complete genome sequence of *Alicyclobacillus* phage MMB025 has been deposited in GenBank under accession number PV686781. Illumina and Oxford Nanopore raw sequencing reads are available under accession numbers SRX31034946 and SRX31034947, respectively, within BioProject PRJNA1359328. The host genome used for mapping is *A. acidoterrestris* DSM 3922^T^ (GenBank accession number CP080467). All other data are provided in the manuscript and Supplementary information files.

## SUPPLEMENTAL MATERIAL

Data S1 spectrum.03747-25-s0001.pdf Full PhageTerm v4.1 report for *Alicyclobacillus* phage MMB025 termini and packaging mode obtained using short reads R1 (forward).

Data S2 spectrum.03747-25-s0002.pdf Full PhageTerm v4.1 report for *Alicyclobacillus* phage MMB025 termini and packaging mode obtained using short reads R2 (reverse).

Data S3 spectrum.03747-25-s0003.pdf Full PhageTerm v4.1 report for *Alicyclobacillus* phage MMB025 termini and packaging mode obtained using ONT long reads.

Fig. S1 spectrum.03747-25-s0004.pdf Morphometric measurements of *Alicyclobacillus* phage MMB025 virions (ImageJ).

Fig. S2 spectrum.03747-25-s0005.docx Representative plaque phenotypes produced by *Alicyclobacillus* phage MMB025 on *Alicyclobacillus* isolates.

Fig. S3 spectrum.03747-25-s0006.docx Summary of PhageTerm analysis of *Alicyclobacillus* phage MMB025 genome termini.

Fig. S4 spectrum.03747-25-s0007.docx Comparative alignment of the *Alicyclobacillus* phage MMB025 genome with the *A. acidoterrestris* DSM 3922^T^ genome.

Table S1 spectrum.03747-25-s0008.docx Detailed strain-level host range of *Alicyclobacillus* phage MMB025.

Table S2 spectrum.03747-25-s0009.xlsx Genome annotation of *Alicyclobacillus* phage MMB025.

ASM does not own the copyrights to Supplemental Material that may be linked to, or accessed through, an article. The authors have granted ASM a non-exclusive, world-wide license to publish the Supplemental Material files. Please contact the corresponding author directly for reuse.
