# Supplementary material for: A novel temperate phage from Alicyclobacillus: first evidence in this genus of genomic identity to a sigK-integrated prophage
Source: Microbiol Spectr. 2026 Apr 3;14(5):e03747-25. doi: 10.1128/spectrum.03747-25 (PMC13141910; doi:10.1128/spectrum.03747-25)
Supplement: Fig. S1 — Morphometric measurements of Alicyclobacillus phage MMB025 virions (ImageJ). [file spectrum.03747-25-s0004.pdf]

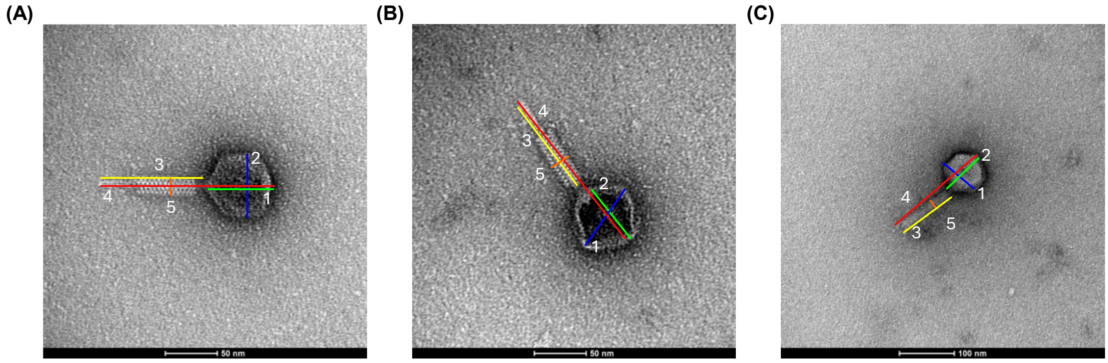

	Figure A	Figure B	Figure C	AVG	STDEV
**Head length (nm)**	61.31	62.38	74.55	66.08	6.00
**Head diameter (nm)**	59.53	61.90	73.72	65.05	6.21
**Tail length (nm)**	95.39	96.48	107.40	99.76	5.42
**Tail diameter (nm)**	17.66	19.65	20.30	19.20	1.12
**Phage virion size (nm)**	163.10	167.05	184.72	171.62	9.40

Figure S1. Morphometric measurements of *Alicyclobacillus* phage MMB025 virions (ImageJ).

Three representative TEM micrographs of phage MMB025 were selected (A to C) and used for measurements, showing virions with a contracted or extended tail. Annotations: (1) head length (green); (2) head diameter (blue); (3) tail length (yellow); (4) virion length (red); (5) tail diameter (orange). Measurements were performed in ImageJ v1.54p; values summarized in the main text. Negative stain: 2% uranyl acetate; microscope at 120 kV. Scale bars: A and B, 50 nm; C, 100 nm.
