# Supplementary material for: A novel temperate phage from Alicyclobacillus: first evidence in this genus of genomic identity to a sigK-integrated prophage
Source: Microbiol Spectr. 2026 Apr 3;14(5):e03747-25. doi: 10.1128/spectrum.03747-25 (PMC13141910; doi:10.1128/spectrum.03747-25)
Supplement: Fig. S2 — Representative plaque phenotypes produced by Alicyclobacillus phage MMB025 on Alicyclobacillus isolates. [file spectrum.03747-25-s0005.docx]

|  | **Panel A** | **Panel B** |
| --- | --- | --- |
| **Lytic phenotype**  **(++)** | 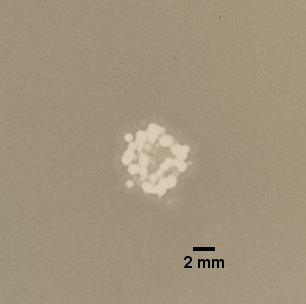 | 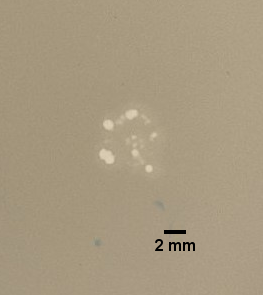 |
| **Medium lysis phenotype (+)** | 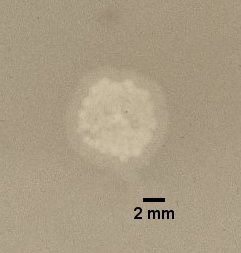 | 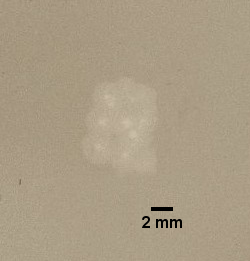 |

**Figure S2 – Representative plaque phenotypes produced by phage MMB025 on *Alicyclobacillus* isolates.** Clear, well‑defined plaques correspond to the complete-lysis phenotype (denoted as “++” in Table 1), whereas turbid or partially cleared plaques correspond to the medium‑lysis phenotype (denoted as “+” in Table 1). Images were obtained from spot assays under standard experimental conditions. Scale bar = 2 mm.
