# Supplementary material for: A novel temperate phage from Alicyclobacillus: first evidence in this genus of genomic identity to a sigK-integrated prophage
Source: Microbiol Spectr. 2026 Apr 3;14(5):e03747-25. doi: 10.1128/spectrum.03747-25 (PMC13141910; doi:10.1128/spectrum.03747-25)
Supplement: Fig. S3 — Summary of PhageTerm analysis of Alicyclobacillus phage MMB025 genome termini. [file spectrum.03747-25-s0006.docx]

**
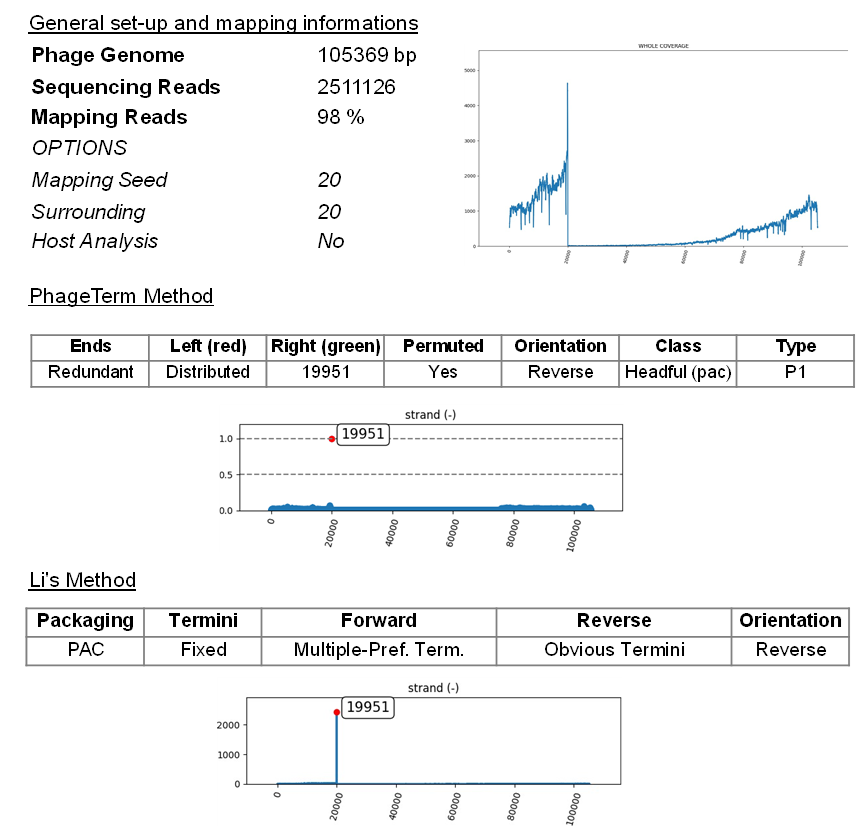
**

**Figure S3. Summary of PhageTerm analysis of *Alicyclobacillus* phage MMB025 genome termini.**

Coverage plot and termini predictions generated by PhageTerm v4.1 using Illumina and ONT reads. A single dominant terminus was detected on the reverse strand, with multiple preferred termini on the forward strand, consistent with a headful (pac-type) packaging mechanism. The predicted pac site (vertical red line) was used to orient the final genome (see Fig. 5). The data shown correspond to PhageTerm predictions obtained using short reads R1 (forward). The same analysis performed with short reads R2 (reverse) and ONT long reads yielded concordant results, all supporting the headful (pac-type) packaging mechanism. Full PhageTerm outputs, including detailed coverage and termini data, are provided in Supplementary Files S1, S2, and S3, corresponding to analyses based on short reads R1, short reads R2, and long reads, respectively.
