# Supplementary material for: A novel temperate phage from Alicyclobacillus: first evidence in this genus of genomic identity to a sigK-integrated prophage
Source: Microbiol Spectr. 2026 Apr 3;14(5):e03747-25. doi: 10.1128/spectrum.03747-25 (PMC13141910; doi:10.1128/spectrum.03747-25)
Supplement: Fig. S4 — Comparative alignment of the Alicyclobacillus phage MMB025 genome with the A. acidoterrestris DSM 3922T genome. [file spectrum.03747-25-s0007.docx]

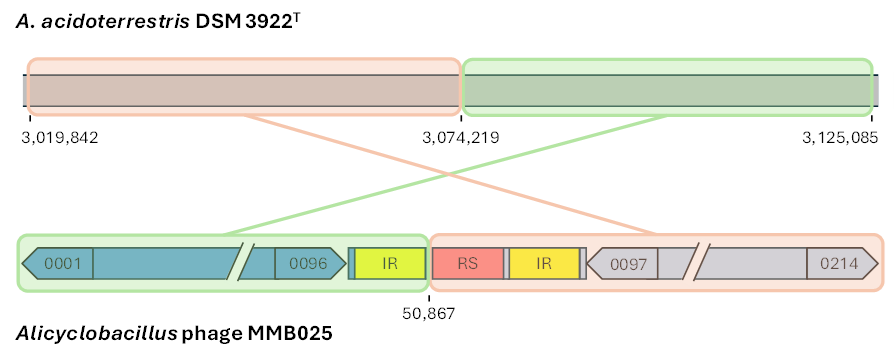


**Figure S4. Comparative alignment of the *Alicyclobacillus* phage MMB025 genome with the *A. acidoterrestris* DSM 3922^T^ genome.**

MAUVE alignment showing extensive nucleotide identity between the complete MMB025 genome and a homologous chromosomal region in *A. acidoterrestris* DSM 3922^T^. Green and red blocks represent collinear and inverted homology regions, respectively. The high level of sequence identity indicates that the MMB025 genome corresponds to a prophage-like region within the *A. acidoterrestris* DSM 3922^T^ chromosome, most likely resulting from a site-specific recombination integration event. Analysis performed using *progressiveMauve* within Geneious Prime v2025.1.2 (http://www.geneious.com).
