# Supplementary material for: A novel temperate phage from Alicyclobacillus: first evidence in this genus of genomic identity to a sigK-integrated prophage
Source: Microbiol Spectr. 2026 Apr 3;14(5):e03747-25. doi: 10.1128/spectrum.03747-25 (PMC13141910; doi:10.1128/spectrum.03747-25)
Supplement: Table S1 — Detailed strain-level host range of Alicyclobacillus phage MMB025. [file spectrum.03747-25-s0008.docx]

**Table S1 – Detailed strain-level host range of *Alicyclobacillus* phage MMB025.** Plaque phenotypes are indicated as follows: ++, clear plaques; +, turbid plaques; -, no plaques detected. Results represent two independent biological replicates.

| **Bacterial strain** | **Phage lytic activity** |
| --- | --- |
| *Alicyclobacillus acidiphilus* DSM 14558^T^ | - |
| *Alicyclobacillus acidocaldarius* DSM 446^T^ | + |
| *A. acidocaldarius* MMB020 | - |
| *Alicyclobacillus acidoterrestris* DSM 3922^T^ | - |
| *A. acidoterrestris* MMB001 | - |
| *A. acidoterrestris* MMB002 | ++ |
| *A. acidoterrestris* MMB003 | ++ |
| *A. acidoterrestris* MMB004 | - |
| *A. acidoterrestris* MMB005 | - |
| *A. acidoterrestris* MMB006 | ++ |
| *A. acidoterrestris* MMB007 | ++ |
| *A. acidoterrestris* MMB008 | - |
| *A. acidoterrestris* MMB009 | - |
| *A. acidoterrestris* MMB010 | - |
| *A. acidoterrestris* MMB011 | ++ |
| *A. acidoterrestris* MMB012 | + |
| *A. acidoterrestris* MMB013 | ++ |
| *A. acidoterrestris* MMB021 | - |
| *A. acidoterrestris* FSL R14-0029 | - |
| *A. acidoterrestris* FSL R14-0046 | - |
| *Alicyclobacillus contaminans* DSM 17975^T^ | - |
| *Alicyclobacillus cycloheptanicus* DSM 4006^T^ | - |
| *A. cycloheptanicus* ATCC 49029 | - |
| *Alicyclobacillus fastidiosus* DSM 17978^T^ | - |
| *Alicyclobacillus fructus* DSM 112018^T^ | - |
| *Alicyclobacillus herbarius* DSM 13609^T^ | - |
| *Alicyclobacillus hesperidum* DSM 11985^T^ | - |
| *Alicyclobacillus kakegawensis* DSM 17979^T^ | - |
| *Alicyclobacillus macrosporangiidus* DSM 17980^T^ | - |
| *Alicyclobacillus mali* DSM 112016^T^ | - |
| *Alicyclobacillus pomorum* DSM 14955^T^ | - |
| *Alicyclobacillus sacchari* DSM 17974^T^ | - |
| *Alicyclobacillus suci* DSM 112017^T^ | - |
| *A. suci* FSL R14-0023 | - |
| *A. suci* FSL R14-0051 | - |
| *Alicyclobacillus tengchongensis* ATCC BAA2134^T^ | - |
| *Alicyclobacillus vulcanalis* ATCC BAA915^T^ | - |
| *Bacillus subtilis* CECT 356 | - |
| *Bacillus cereus* BGSC 6A1 | - |
| *Escherichia coli* ATCC 25922 | - |
| *Geobacillus stearothermophilus* DSM 22^T^ | - |
| *Staphylococcus aureus* ATCC 6538 | - |
